# Supplementary material for: End Sequence Analysis Toolkit (ESAT) expands the extractable information from single-cell RNA-seq data
Source: Genome Res. 2016 Oct;26(10):1397–410. doi: 10.1101/gr.207902.116 (PMC5052061; doi:10.1101/gr.207902.116)
Supplement: Supplemental Material [file supp_gr.207902.116_Supp_Materials.tar.gz › ESAT-esat_v0.1/doc/html/classumms_1_1core_1_1model_1_1_alignment_model-members.html]

ESAT: Member List


ESAT


- Main Page
- Related Pages
- Packages
- Classes
- Files

- Class List
- Class Index
- Class Hierarchy
- Class Members

All Classes Namespaces Files Functions Variables Enumerator Pages

- umms
- core
- model
- AlignmentModel

umms.core.model.AlignmentModel Member List

This is the complete list of members for umms.core.model.AlignmentModel, including all inherited members.

|  |  |  |
| --- | --- | --- |
| addFilter(Predicate< Alignment > filter) | umms.core.model.AlignmentModel |  |
| addFilters(Collection< Predicate< Alignment >> filters) | umms.core.model.AlignmentModel |  |
| AlignmentModel(String bamFile, CoordinateSpace coordinateSpace, Collection< Predicate< Alignment >> readFilters, boolean readOrCreatePairedEndBam) | umms.core.model.AlignmentModel |  |
| AlignmentModel() | umms.core.model.AlignmentModel |  |
| AlignmentModel(String bamFile, CoordinateSpace coordinateSpace, Collection< Predicate< Alignment >> readFilters, boolean readOrCreatePairedEndBam, TranscriptionRead transcriptionRead) | umms.core.model.AlignmentModel |  |
| AlignmentModel(String bamFile, CoordinateSpace coordinateSpace, Collection< Predicate< Alignment >> readFilters, boolean readOrCreatePairedEndBam, TranscriptionRead transcriptionRead, boolean fragment) | umms.core.model.AlignmentModel |  |
| AlignmentModel(String bamFile, CoordinateSpace coordinateSpace, Collection< Predicate< Alignment >> readFilters, boolean readOrCreatePairedEndBam, TranscriptionRead transcriptionRead, String maskedRegionFile) | umms.core.model.AlignmentModel |  |
| AlignmentModel(String bamFile, CoordinateSpace coordinateSpace, Collection< Predicate< Alignment >> readFilters, boolean readOrCreatePairedEndBam, TranscriptionRead transcriptionRead, boolean fragment, String maskedRegionFile) | umms.core.model.AlignmentModel |  |
| AlignmentModel(String bamFile, CoordinateSpace coordinateSpace, Collection< Predicate< Alignment >> readFilters) | umms.core.model.AlignmentModel |  |
| AlignmentModel(String bamFile, CoordinateSpace coordinateSpace, Collection< Predicate< Alignment >> readFilters, TranscriptionRead transcriptionRead) | umms.core.model.AlignmentModel |  |
| AlignmentModel(String bamFile, CoordinateSpace coordinateSpace, boolean readOrCreatePairedEndBam) | umms.core.model.AlignmentModel |  |
| AlignmentModel(String bamFile, CoordinateSpace coordinateSpace, boolean readOrCreatePairedEndBam, TranscriptionRead transcriptionRead) | umms.core.model.AlignmentModel |  |
| AlignmentModel(String bamFile, CoordinateSpace coordinateSpace) | umms.core.model.AlignmentModel |  |
| AlignmentModel(String bamFile, CoordinateSpace coordinateSpace, TranscriptionRead transcriptionRead) | umms.core.model.AlignmentModel |  |
| computeGlobalStats() | umms.core.model.AlignmentModel |  |
| containsReference(String refName) | umms.core.model.AlignmentModel |  |
| coordinateSpace | umms.core.model.AlignmentModel | protected |
| getAverageCountPerPosition(Gene gene) | umms.core.model.AlignmentModel |  |
| getAverageCountPerPositionWithThreshold(Gene gene, int minCount) | umms.core.model.AlignmentModel |  |
| getBamFile() | umms.core.model.AlignmentModel |  |
| getBasesCovered(Annotation region, boolean fullyContained) | umms.core.model.AlignmentModel |  |
| getBasesCovered(Annotation region) | umms.core.model.AlignmentModel |  |
| getCoordinateSpace() | umms.core.model.AlignmentModel |  |
| getCount(Annotation window, boolean fullyContained) | umms.core.model.AlignmentModel |  |
| getCount() | umms.core.model.AlignmentModel |  |
| getCount(CloseableIterator< AlignmentCount > iter) | umms.core.model.AlignmentModel |  |
| getCount(Annotation window) | umms.core.model.AlignmentModel |  |
| getCountExcludingRegion(Annotation region, Annotation excluded) | umms.core.model.AlignmentModel |  |
| getCountsPerPosition(Gene gene) | umms.core.model.AlignmentModel |  |
| getCountsStrandedPerPosition(Annotation gene) | umms.core.model.AlignmentModel |  |
| getCountStrandedExcludingRegion(Annotation region, Annotation excluded) | umms.core.model.AlignmentModel |  |
| getGlobalCount() | umms.core.model.AlignmentModel |  |
| getGlobalCountReferenceSeqs() | umms.core.model.AlignmentModel |  |
| getGlobalLambda() | umms.core.model.AlignmentModel |  |
| getGlobalLength() | umms.core.model.AlignmentModel |  |
| getGlobalNumReads() | umms.core.model.AlignmentModel |  |
| getGlobalNumReadsReferenceSeqs() | umms.core.model.AlignmentModel |  |
| getGlobalPairedFragments() | umms.core.model.AlignmentModel |  |
| getIntronCounts(Annotation intron) | umms.core.model.AlignmentModel |  |
| getMedianReadSize(Annotation region, CoordinateSpace coord, int maxSize, int numBins, boolean fullyContained) | umms.core.model.AlignmentModel |  |
| getMedianReadSize(Annotation region, CoordinateSpace coord, int maxSize, int numBins) | umms.core.model.AlignmentModel |  |
| getOverlappingAnnotations(Annotation region, boolean fullyContained) | umms.core.model.AlignmentModel |  |
| getOverlappingReadCounts(Annotation region, boolean fullyContained) | umms.core.model.AlignmentModel |  |
| getOverlappingReadReplicateCounts(Annotation region, boolean fullyContained) | umms.core.model.AlignmentModel |  |
| getOverlappingReads(String chr) | umms.core.model.AlignmentModel |  |
| getOverlappingReads(Annotation region, boolean fullyContained) | umms.core.model.AlignmentModel |  |
| getOverlappingSplicedReads(Annotation region, boolean fullyContained) | umms.core.model.AlignmentModel |  |
| getOverlappingSplicedReads(String chr) | umms.core.model.AlignmentModel |  |
| getPeak(Annotation parent, Annotation region, boolean fullyContained) | umms.core.model.AlignmentModel |  |
| getPermutedAnnotations(Annotation region) | umms.core.model.AlignmentModel |  |
| getPositionCountList(Gene gene) | umms.core.model.AlignmentModel |  |
| getReadIterator() | umms.core.model.AlignmentModel |  |
| getReadIterator(Annotation region) | umms.core.model.AlignmentModel |  |
| getReadSizeDistribution(CoordinateSpace coord, int maxSize, int numBins) | umms.core.model.AlignmentModel |  |
| getReadSizeDistribution(Annotation region, CoordinateSpace coord, int maxSize, int numBins) | umms.core.model.AlignmentModel |  |
| getReadSizeDistribution(Annotation region, CoordinateSpace coord, int maxSize, int numBins, boolean fullyContained) | umms.core.model.AlignmentModel |  |
| getRefSequenceCounts(String refName) | umms.core.model.AlignmentModel |  |
| getRefSequenceLambda(String refName) | umms.core.model.AlignmentModel |  |
| getRefSequenceLength(String refName) | umms.core.model.AlignmentModel |  |
| getRefSequenceLengths() | umms.core.model.AlignmentModel |  |
| getSplicedCount(Alignments alignments, boolean fullyContained) | umms.core.model.AlignmentModel |  |
| getStrand() | umms.core.model.AlignmentModel |  |
| isValid(Alignment read) | umms.core.model.AlignmentModel |  |
| iterator() | umms.core.model.AlignmentModel |  |
| removeFilter(Predicate< Alignment > readFilter) | umms.core.model.AlignmentModel |  |
| scan(Annotation region, int windowSize, int overlap) | umms.core.model.AlignmentModel |  |
| size() | umms.core.model.AlignmentModel |  |
| windowIterator(int windowSize, int overlap) | umms.core.model.AlignmentModel |  |


---

Generated on Fri Aug 15 2014 15:11:11 for ESAT by  

 1.8.7
